# Cost-effectiveness of aficamten for obstructive hypertrophic cardiomyopathy: a model-based analysis from the perspective of the Chinese healthcare system

**DOI:** 10.3389/fphar.2026.1938259

**Published:** 2026-10-07

**Authors:** Feng Zhai, Hongyang Li, Chaolan Jiang, Jianpeng Mao, Jingjing Chen, Caicong You

**Affiliations:** 1 Department of Pharmacy, Anhui Wannan Rehabilitation Hospital (Wuhu Fifth People’s Hospital), Wuhu, Anhui, China; 2 Department of Pharmacy, The First Affiliated Hospital of Anhui Medical University, Hefei, Anhui, China; 3 School of Pharmacy, Fujian Medical University, Fuzhou, Fujian, China

**Keywords:** aficamten, cost-effectiveness analysis, Markov model, obstructive hypertrophic cardiomyopathy, pharmacoeconomics

## Abstract

**Background:**

To evaluate the cost-effectiveness of aficamten plus standard of care (SoC) versus SoC alone for patients with symptomatic obstructive hypertrophic cardiomyopathy (oHCM) from the perspective of the Chinese healthcare system.

**Methods:**

A four-state Markov model based on the phase III SEQUOIA-HCM trial was developed to simulate lifetime outcomes. The first 24 weeks were modeled using trial data, followed by 4-week cycles for long-term extrapolation. Based on FOREST-HCM, the incremental treatment effect of aficamten was assumed to persist through week 96 and then linearly wane to complete loss by year 3. Costs and health outcomes were discounted at 4.5% annually. Model uncertainty was assessed using one-way sensitivity analysis, probabilistic sensitivity analysis, and scenario analyses.

**Results:**

Total costs were US$143,281.22 with aficamten plus SoC and US$96,509.03 with SoC alone, with 9.696 and 7.643 QALYs, respectively. Aficamten plus SoC yielded an incremental cost of US$46,772.19 and 2.054 QALYs, corresponding to an ICER of US$22,775.17/QALY, below the WTP threshold of US$29,399.70/QALY. The probability of being cost-effective was 76.6% at the WTP threshold. Scenario analyses showed ICERs of US$19,351.77/QALY when only short-term effects were considered, US$43,752.42/QALY when treatment effects disappeared in year 5, and US$81,220.72/QALY when they disappeared in year 10. Price reductions of 22.0% and 50.5%, respectively, were required in the latter two scenarios. With a permanent discontinuation probability of 0.131% per 4-week cycle, the ICER was US$23,888.94/QALY.

**Conclusion:**

From the Chinese healthcare system perspective, aficamten plus SoC was cost-effective versus SoC alone for symptomatic oHCM under the base-case assumptions.

## Introduction

Hypertrophic cardiomyopathy (HCM) is a myocardial disorder characterized by left ventricular hypertrophy caused by pathogenic variants in genes encoding sarcomeric proteins or by an unidentified etiology. It should be distinguished from ventricular wall thickening attributable to other cardiovascular, systemic, or metabolic diseases (National Center for Cardiovascular Diseases Cardiomyopathy Alliance, 2025). HCM is the most common monogenic inherited cardiac disease, with an estimated prevalence of approximately 1 in 500 adults, and occurs across sexes, ethnicities, and age groups worldwide (Ommen et al., 2024). The clinical presentation of HCM is highly heterogeneous. Many patients remain asymptomatic for long periods, whereas others experience dyspnea, chest pain, palpitations, fatigue, impaired exercise tolerance, or syncope. These manifestations are largely driven by the complex pathophysiology of HCM. Approximately 30%–40% of patients with HCM have oHCM, in which systolic protrusion of the hypertrophied septum into the left ventricular outflow tract (LVOT), together with systolic anterior motion of the mitral valve leaflet, contributes to dynamic obstruction and limits cardiac output (Li and Wang, 2023).

Conventional pharmacologic therapies, including beta-blockers and non-dihydropyridine calcium channel blockers, are recommended as first-line treatments for oHCM (National Center for Cardiovascular Diseases Cardiomyopathy Alliance, 2025). These agents alleviate symptoms primarily by slowing heart rate and reducing myocardial contractility; however, they do not directly address the underlying sarcomeric dysfunction. Some patients therefore continue to experience symptoms and may require invasive septal reduction therapy (SRT) (Dybro et al., 2021). Although SRT can effectively relieve LVOT obstruction, it is associated with procedural risks, potential complications, and substantial technical requirements, and is not appropriate for all patients. Safe and reliable pharmacologic options that improve patient symptoms and functional status with acceptable tolerability remain an important unmet clinical need (Elliott, 2020). Mavacamten, the first-in-class cardiac myosin inhibitor (CMI), has been shown to improve exercise capacity and symptoms in patients with oHCM (Braunwald et al., 2023; Desai et al., 2022; Olivotto et al., 2020).

Aficamten is a second-generation small-molecule CMI. It binds specifically to the ATPase domain of the cardiac myosin head and dose-dependently inhibits actin-myosin cross-bridge formation. By reducing the number of recruitable active cross-bridges, aficamten decreases myocardial contractility and improves diastolic function (Ogieuhi et al., 2025). Its pharmacokinetic profile permits individualized dose titration every 2 weeks, which distinguishes it from mavacamten (Malik et al., 2022; Sharpe et al., 2023). In the phase 3 SEQUOIA-HCM trial (NCT05186818), aficamten significantly improved peak oxygen uptake (pVO2; the primary endpoint) compared with placebo after 24 weeks of treatment. Resting and Valsalva LVOT gradients decreased by approximately 37 mmHg and 50 mmHg, respectively, and both New York Heart Association (NYHA) functional class and Kansas City Cardiomyopathy Questionnaire Clinical Summary Score (KCCQ-CSS) improved significantly. Regarding safety, transient reductions in left ventricular ejection fraction (LVEF) below 50% occurred in 3.5% of patients, while no patient experienced LVEF below 40% or drug-related heart failure events (Maron et al., 2024).

Additional evidence from REDWOOD-HCM and MAPLE-HCM further supports the efficacy of aficamten in reducing LVOT gradients and improving exercise capacity and symptoms, and the FOREST-HCM long-term extension study suggests sustained efficacy and safety (Tower-Rader et al., 2026; Maron et al., 2023; Garcia-Pavia et al., 2025). The 2023 European Society of Cardiology guideline and the 2024 American Heart Association/American College of Cardiology (AHA/ACC) guideline both emphasize stepwise treatment, SRT, and novel CMIs in the management of symptomatic oHCM, providing a clinical basis for the treatment pathway used in this study (Ommen et al., 2024; Arbelo et al., 2023). Aficamten was approved in China in December 2025 for the treatment of adults with NYHA class II-III oHCM to improve exercise capacity and symptoms (Cytokinetics Incorporated, 2023). Currently, aficamten has not yet been included in China’s National Reimbursement Drug List, and its market price is higher than that of comparable CMIs covered by national health insurance. Its clinical use may therefore impose substantial drug expenditures, underscoring the need for a pharmacoeconomic evaluation to inform its rational clinical use and reimbursement decision-making. However, head-to-head randomized controlled trials comparing aficamten with other CMIs are currently lacking, and evidence directly comparing their relative clinical effectiveness and economic value remains limited. Accordingly, based on clinical data from the SEQUOIA-HCM trial, this study developed a four-state Markov model to evaluate, from the perspective of the Chinese healthcare system, the cost-effectiveness of aficamten plus conventional SoC versus SoC alone in patients with oHCM, with the aim of providing pharmacoeconomic evidence to inform the clinical use, reimbursement decisions, and broader healthcare decision-making related to aficamten.

## Materials and methods

This study developed a pharmacoeconomic model using Microsoft Office 365 Excel to evaluate the cost-effectiveness of aficamten plus SoC versus SoC alone for the treatment of adults with symptomatic oHCM from the perspective of the Chinese healthcare system. The model structure was based on a previously published pharmacoeconomic evaluation framework for oHCM (Cotté et al., 2026). As this study was a pharmacoeconomic modeling analysis based on published literature and publicly available data and did not involve human participants, additional informed consent was not required, nor was approval from an institutional research ethics committee necessary. This study was performed according to the Consolidated Health Economic Evaluation Reporting Standards 2022 (CHEERS 2022) (Husereau et al., 2022). Detailed information is provided in Supplementary Table S1.

### Model structure

A four-state Markov model was used, comprising four mutually exclusive health states: NYHA class I, NYHA class II, NYHA class III/IV, and death (Figure 1). NYHA functional class was used to define health states for three reasons. First, NYHA class is a widely used clinical standard for assessing heart failure severity in guidelines and routine practice (National Center for Cardiovascular Diseases Cardiomyopathy Alliance, 2025; Ommen et al., 2024). Second, NYHA class is closely associated with quality of life and mortality, making it suitable for modeling disease natural history. Third, because patients with NYHA class IV represented only 0.7% of the SEQUOIA-HCM population (Maron et al., 2024), NYHA classes III and IV were combined to reduce uncertainty in parameter estimation. This structure is consistent with published cost-effectiveness models in oHCM (Cotté et al., 2026; Qian et al., 2026).

**FIGURE 1:**
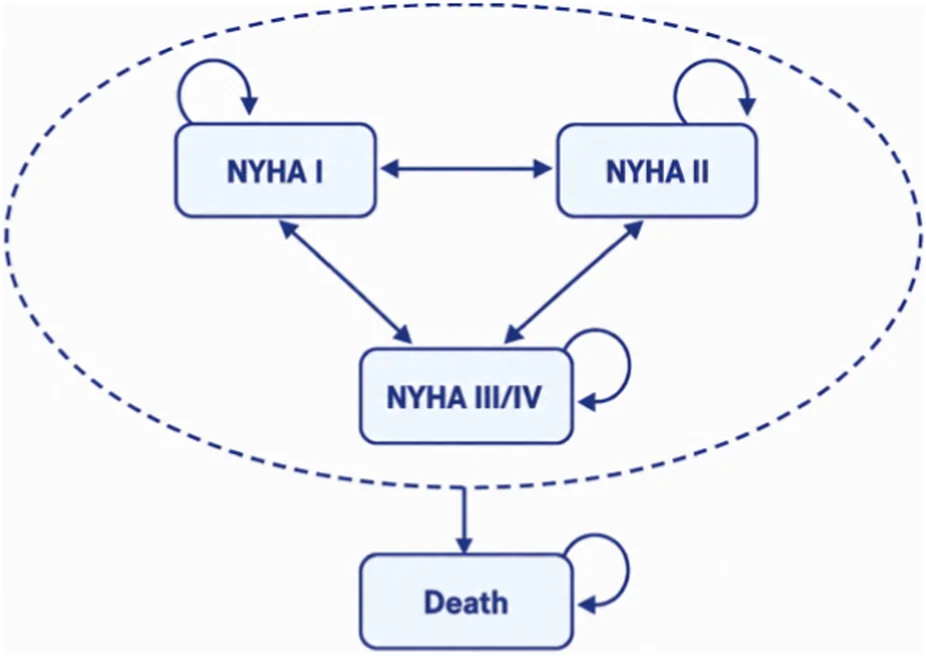
Markov model structure with NYHA class I, NYHA class II, NYHA class III/IV, and death health states.

### Simulation settings and target population

The starting age of the model cohort was set at 59.1 years, corresponding to the median age of participants in the SEQUOIA-HCM trial (Maron et al., 2024). A lifetime time horizon was adopted to fully capture the long-term clinical and economic consequences of oHCM as a chronic progressive disease. The model consisted of two phases: a short-term phase and a long-term extrapolation phase. The first 24 weeks represented the short-term trial phase and were primarily informed by data from the SEQUOIA-HCM trial. After week 24, patients entered the long-term extrapolation phase, with a fixed cycle length of 4 weeks.

The model population was consistent with the intention-to-treat population of the SEQUOIA-HCM trial, whose demographic characteristics were considered comparable to those of Chinese patients with oHCM seeking medical care. According to the trial data, the baseline NYHA class distribution in both treatment groups was 0% for NYHA class I, 75.9% for NYHA class II, and 24.1% for NYHA class III/IV (Maron et al., 2024). Patients entered the model according to this baseline distribution.

### Treatment strategies and comparators

According to the design of the SEQUOIA-HCM trial and recommendations in Chinese clinical practice, patients in the aficamten group received aficamten plus SoC, whereas those in the SoC group received SoC alone. Background SoC therapy was weighted according to the proportions of β-blockers and non-dihydropyridine calcium channel blockers observed in the SEQUOIA-HCM trial (Maron et al., 2024). Because disopyramide tablets are no longer marketed in China and are rarely used in current clinical practice, disopyramide was excluded from the analysis.

For eligible patients who remained in NYHA class III/IV despite long-term treatment, SRT was incorporated into the model as a subsequent treatment option, consistent with recommendations from the 2024 AHA/ACC guideline and the Chinese Guidelines for Comprehensive Management of Cardiomyopathy (2025) (1,2).

### Transition probabilities

The model comprised a short-term trial phase and a long-term extrapolation phase, with NYHA health-state transitions specified separately for each phase. The short-term phase extended from baseline to week 24. Although clinical assessment visits in the SEQUOIA-HCM trial were scheduled at weeks 2, 4, 6, 8, 12, 16, 20, and 24, the interim visits were primarily used for dose titration, efficacy and safety monitoring, and estimation of related healthcare resource utilization, and the NYHA state-transition matrix was not repeatedly applied at these visits (Maron et al., 2024). The short-term NYHA transition matrix was applied to surviving patients over the 24-week period to reflect the observed improvements in NYHA functional class during the 24-week SEQUOIA-HCM trial.

The SEQUOIA-HCM trial showed that, at week 24, the proportions of patients with an improvement of at least one NYHA class were 58.5% in the aficamten group and 24.3% in the SoC group. Because publicly available data did not provide individual-level transition matrices stratified by baseline NYHA class, this study assumed that the same probability of improvement applied across different baseline NYHA health states. Specifically, patients with baseline NYHA class II who improved by at least one class were assumed to transition to NYHA class I, whereas patients with baseline NYHA class III/IV who improved by at least one class were assumed to transition to NYHA class II; patients without improvement were assumed to remain in their original NYHA state. Accordingly, the week-24 transition probabilities for NYHA II→I and NYHA III/IV→II were both set at 0.585 in the aficamten group and 0.243 in the SoC group. The uncertainty associated with this assumption was further examined in sensitivity analyses. After week 24, patients entered the long-term Markov extrapolation phase, using a fixed 4-week cycle length. The transition probabilities for the long-term natural disease course were derived from a previously published Chinese pharmacoeconomic study of oHCM, with the probability of natural deterioration to the next adjacent NYHA class set at 0.0034 per 4-week cycle (Qian et al., 2026). Because NYHA classes III and IV were combined into a single health state in the present model, further deterioration from NYHA class III to class IV was considered to occur within the NYHA III/IV state. The persistence and waning of the long-term treatment effect of aficamten were modeled according to the base-case assumptions described below. Permanent discontinuation was evaluated separately in Scenario 2. The transition probabilities between NYHA health states are presented in Table 1.

**TABLE 1:** Transition probabilities between NYHA classes.

NYHA class	NYHA I	NYHA II	NYHA III/IV
Short-term transition probabilities (<24 weeks)			
Aficamten + SoC			
NYHA I	1.000	0.000	0.000
NYHA II	0.585	0.415	0.000
NYHA III/IV	0.000	0.585	0.415
SoC			
NYHA I	1.000	0.000	0.000
NYHA II	0.243	0.757	0.000
NYHA III/IV	0.000	0.243	0.757
Long-term transition probabilities (>24 weeks)			
NYHA I	0.9966	0.0034	0.0000
NYHA II	0.0000	0.9966	0.0034
NYHA III/IV	0.0000	0.0000	1.0000

NYHA,new york heart association; SoC, standard of care; NYHA-to-NYHA, transition probabilities are conditional on survival; age- and NYHA-specific mortality probabilities are applied separately in each model cycle.

### Long-term treatment-effect extrapolation assumptions

Given that the primary efficacy assessment period of the SEQUOIA-HCM randomized controlled trial was 24 weeks, the long-term model extrapolated the persistence of aficamten treatment effects using data from the FOREST-HCM extension study. In the base-case analysis, evidence from FOREST-HCM was used only to support maintenance of the treatment effect of aficamten through the first 96 weeks, without assuming that the observed benefit would persist indefinitely beyond the available follow-up period. After week 96, the model assumed that the incremental treatment effect of aficamten relative to SoC would gradually wane over time and completely disappear by year 3 of treatment. Once the incremental treatment effect had fully disappeared, patients in the aficamten group were assumed to receive no further additional benefit in terms of NYHA class improvement, and subsequent disease progression was simulated using the long-term natural disease progression transition probabilities applied to the SoC group.

### Mortality

The base-case analysis assumed that no deaths occurred during the first 24-week short-term trial phase. From the long-term Markov phase beginning after week 24, the risk of all-cause mortality was incorporated into each 4-week cycle. Baseline mortality risk was derived from age-specific all-cause mortality rates in the general Chinese population obtained from the Seventh National Population Census of China (2020) (Office of the Leading Group, 2022). Because the model did not distinguish background mortality by sex, age-specific mortality rates for the overall population were used. Mortality risks across NYHA functional classes were further adjusted using all-cause mortality hazard ratios reported by Wang et al. from a real-world cohort of patients with oHCM (Wang et al., 2023). Using NYHA class I as the reference, the mortality HRs for patients in NYHA classes I, II, and III/IV were set at 1.00, 1.80, and 4.94, respectively (Wang et al., 2016). To align mortality estimates with the 4-week model cycle, the annual all-cause mortality probability q_a_ for individuals aged a was first converted to a continuous-time mortality hazard:

$$h_{a}=-\ln\left(1-q_{a}\right)$$

The corresponding mortality hazard ratio HRi was then applied according to NYHA health state i, and the adjusted mortality hazard was converted to a 4-week cycle mortality probability as follows:

$$P_{a,i}=1-\exp\left[-{\text{HR}}_{i}\times \left(-\ln\left(1-q_{a}\right)\right)\times 4/52\right]$$

where HR_i​_was 1.00, 1.80, and 4.94 for NYHA classes I, II, and III/IV, respectively. Death was modeled as an absorbing state, such that no further health-state transitions occurred after a patient entered the death state. The age-specific natural mortality rates are presented in Table 2, and the mortality hazard ratios and related parameters are presented in Table 3.

**TABLE 2:** Age-specific natural mortality rates.

Age group, years	Mortality rate (per mille)
55–59	7.60
60–64	13.23
65–69	21.90
70–74	46.96
75–79	90.62
80–84	169.99
85–89	288.67
90+	455.32

Mortality rates are age-specific natural mortality rates from the Seventh National Population Census of China and are reported per mille, corresponding to deaths per 1,000 population.

**TABLE 3:** Clinical input parameters and sources.

Parameter	Base case	Lower bound	Upper bound	Distribution	Source
Clinical parameters					
Initial health state distribution					
NYHA class I	0	0	0	NA	12
NYHA class II	0.759	-	-	NA	12
NYHA class III/IV	0.241	-	-	NA	12
Week-24 NYHA improvement probability					
Aficamten + SoC: NYHA II→I	0.585	0.468	0.702	Beta	12
Aficamten + SoC: NYHA III/IV→II	0.585	0.468	0.702	Beta	12
SoC: NYHA II→I	0.243	0.194	0.292	Beta	12
SoC: NYHA III/IV→II	0.243	0.194	0.292	Beta	12
Long-term natural NYHA deterioration, per 4-week cycle	0.0034	0.00272	0.00408	Beta	20
Mortality hazard ratio					
NYHA class I	1.00	1.0	1.0	NA	22
NYHA class II	1.80	1.44	2.16	Log-normal	22
NYHA class III/IV	4.94	3.95	5.93	Log-normal	22
Treatment-related adverse events					
LVEF <50%,first 24 weeks (Aficamten + SoC)	0.035	0.028	0.042	Beta	12
Atrial fibrillation, first 24 weeks	0.007	0.0056	0.0084	Beta	12
Short-term discontinuation due to AEs	0.007	0.0056	0.0084	Beta	12
Scenario analysis parameters					
Permanent discontinuation of aficamten	0.00131	0.00105	0.00157	Beta	13
Subsequent treatment					
SRT in NYHA class III/IV, per 4-week cycle	0.0337	0.027	0.040	Beta	20,25
Improvement from NYHA III/IV to NYHA II following SRT	0.371	0.297	0.445	Beta	20
Utilities					
NYHA class I	0.91	0.728	1	Beta	20
NYHA class II	0.85	0.680	1	Beta	20
NYHA class III/IV	0.70	0.560	0.84	Beta	20
Atrial fibrillation disutility	0.19	0.228	0.152	NA	24
Costs (USD)					
Drug acquisition cost per week					
Aficamten, per 5-mg tablet	29.50	23.60	35.40	Gamma	26
Metoprolol tablets,per week	0.233	0.187	0.280	Gamma	26
Verapamil tablets,per week	6.04	4.83	7.25	Gamma	26
Aficamten monitoring costs					
Routine monitoring, weeks 0–24	230.09	184.07	276.11	Gamma	28
Long-term monitoring, per 4-week cycle	11.80	9.44	14.16	Gamma	28
Additional monitoring when LVEF <50%, per event	38.35	30.68	46.02	Gamma	28
Atrial fibrillation management, per case	6,805.68	5,444.54	8,166.81	Gamma	24
Annual direct medical costs					
NYHA class I	2,013.94	1,611.15	2,416.72	Gamma	20
NYHA class II	7,997.02	6,397.61	9,596.42	Gamma	20
NYHA class III/IV	19,916.60	15,933.28	23,899.92	Gamma	20
SRT, per procedure	991.27	793.02	1,189.53	Gamma	26
Discount rate	4.5%	0%	5%	NA	27

AE, adverse event; NYHA, new york heart association; SRT, septal reduction therapy.

### Treatment-related adverse events and subsequent treatment

Treatment-related adverse events (TRAEs) were considered in the model. Based on the adverse events observed in the SEQUOIA-HCM trial (Maron et al., 2024), only grade 3–4 AEs were included in the cost and utility analyses. In the base-case analysis, atrial fibrillation (AF) was the only treatment-related adverse event included, as it represents a clinically important complication requiring long-term management. The cost of AF management was set at US$6,805.68 per case, based on a published Chinese economic study of atrial fibrillation (Li et al., 2025). SRT was incorporated into the model as a subsequent treatment during the long-term simulation phase. SRT was modeled from the long-term Markov phase beginning after week 24, with no additional SRT events assumed during the short-term randomized controlled trial phase. In the base-case analysis, only patients who were alive, in NYHA class III/IV, and had not previously undergone SRT were eligible to enter the SRT treatment pathway. SRT utilization rates were derived from a survey of Chinese cardiologists treating patients with oHCM conducted at ISPOR Europe 2024. The survey reported annual proportions of patients undergoing SRT of 16.37%, 21.40%, 32.80%, and 39.26% for NYHA classes I, II, III, and IV, respectively (Tian et al., 2024). Given that NYHA classes III and IV were combined into a single health state in this study, and with reference to a previously published Chinese pharmacoeconomic model of oHCM, the base-case analysis assumed an annual conditional probability of SRT of 36.0% among patients in NYHA class III/IV. The corresponding 4-week cycle probability was 3.37% (Qian et al., 2026). This conditional probability was applied equally to eligible patients in both treatment groups.

SRT was modeled as a one-time clinical event, and patients who underwent SRT were no longer subject to the probability of receiving SRT in subsequent cycles. Based on previous real-world studies and Chinese pharmacoeconomic models of oHCM, 37.1% of patients were assumed to improve from NYHA class III/IV to NYHA class II following SRT, while the remaining 62.9% remained in NYHA class III/IV. To avoid overestimating treatment benefits, the model did not allow patients to transition directly from NYHA class III/IV to NYHA class I during the cycle in which SRT was performed. After postoperative redistribution across NYHA health states, patients continued to be simulated according to the long-term natural disease progression transition probabilities of the SoC group.

The base-case analysis did not include the background probability of permanent aficamten discontinuation; however, patients undergoing SRT were assumed to discontinue aficamten as part of the predefined subsequent-treatment pathway. Therefore, from the subsequent cycle onward, aficamten acquisition costs and aficamten-specific monitoring costs were no longer accrued, while patients continued to receive SoC. The cost of SRT was applied as a one-time cost in the cycle in which the procedure was performed. Based on publicly available Chinese medical pricing data from Yaozh.com, the cost of SRT was set at US$991.27 per procedure (Yaozhi.com, 2023).

### Health utilities and costs

According to the China Guidelines for Pharmacoeconomic Evaluations (2025) (Wu and Liu, 2025), health utilities used to estimate quality-adjusted life-years (QALYs) should be based on Chinese population preferences when possible. Because published SEQUOIA-HCM data did not provide NYHA-state-specific EQ-5D-5L utility values suitable for model parameterization, utility values were obtained from a published Chinese oHCM economic model (Qian et al., 2026). Base-case utility values were 0.91 for NYHA class I, 0.85 for NYHA class II, and 0.70 for NYHA class III/IV. Utility values were assumed to be the same across treatment groups within each NYHA class and to decline with worsening NYHA class. Therefore, a specific disutility associated with grade 3 or higher AEs was included. Based on SEQUOIA-HCM, grade 3 or higher AEs were represented primarily by atrial fibrillation, and a disutility of −0.19 was obtained from published literature (Li et al., 2025).

This study was conducted from the perspective of the Chinese healthcare system and included only direct medical costs, comprising drug acquisition costs, aficamten-specific monitoring costs, costs of managing treatment-related adverse events, disease management costs by NYHA functional class, and SRT costs. All costs were expressed in 2025 US dollars. Drug prices were obtained from a publicly available Chinese pharmaceutical pricing database (Yaozhi.com, 2023).

The acquisition cost of aficamten was calculated according to the dose-titration regimen in the SEQUOIA-HCM trial and the proportion of patients receiving each dose. The currently available formulation of aficamten in China is 5 mg × 28 tablets, priced at US$29.50 per tablet. Treatment was initiated at 5 mg/day and gradually titrated according to treatment response to a maximum of 20 mg/day; doses of 10, 15, and 20 mg/day were calculated based on the use of 2, 3, and 4 tablets of the 5-mg formulation per day, respectively. Based on the dose distribution across different stages of the trial, the weighted average weekly drug costs during weeks 0–2, 2–4, 4–6, and 6–8 were US$206.49, US$405.60, US$578.17, and US$678.47, respectively. From week 8 onward, the average weekly drug cost, calculated according to the maintenance-phase dose distribution, was US$667.37.

SoC drug costs were weighted according to the background treatment composition observed in the SEQUOIA-HCM trial, with the utilisation proportions of metoprolol and verapamil being 61.35% and 28.72%, respectively. Aficamten-specific monitoring costs were applied only to the aficamten plus SoC group and were calculated separately from routine disease management costs associated with NYHA functional class. Each monitoring visit included transthoracic color Doppler echocardiography and a specialist cardiovascular outpatient assessment, at a cost of US$38.35 per visit. Assessment of left ventricular ejection fraction (LVEF) was included as part of the echocardiographic examination and was therefore not costed separately (Anhui Provinci al Healthcare Security Administration, 2025). The monitoring frequency was determined according to the clinical monitoring schedule during treatment initiation, dose titration, and long-term therapy. For patients who experienced LVEF <50%, an additional monitoring cost of US$38.35 per visit was applied. After permanent discontinuation of aficamten, no further aficamten-specific monitoring costs were accrued. With respect to adverse events, the analysis included only events considered clinically and economically relevant, namely, those that could result in additional healthcare resource utilization, treatment modification, or permanent treatment discontinuation, rather than all adverse events reported in the trial. The economic impact of LVEF <50% was captured through additional monitoring costs, while the management cost of atrial fibrillation was set at US$6,805.68 per case. Aficamten-related permanent discontinuation was modeled by discontinuing subsequent aficamten acquisition and aficamten-specific monitoring costs and switching patients to SoC treatment. The annual direct medical costs associated with different NYHA functional classes were US$2,013.94 for NYHA class I, US$7,997.02 for NYHA class II, and US$19,916.60 for NYHA class III/IV, reflecting healthcare resource utilization related to routine outpatient care, hospitalization, and other disease management. SRT was modeled as a one-time event at a cost of US$991.27 per procedure. All key cost parameters and the ranges used in sensitivity analyses are presented in Table 3.

### Discounting

In accordance with the China Guidelines for Pharmacoeconomic Evaluations (2025) (Wu and Liu, 2025), both costs and health outcomes were discounted at an annual rate of 4.5% in the base-case analysis. In one-way sensitivity analysis, the discount rate was varied from 0% to 5% to assess the robustness of the results. Clinical input parameters are summarized in Table 3.

## Model outcomes

The primary model outcomes included total costs, QALYs, and the incremental cost-effectiveness ratio (ICER). According to the Statistical Communique of the People’s Republic of China on National Economic and Social Development in 2025, China’s gross domestic product (GDP) *per capita* in 2025 was $14,699.85 (National Bureau of Statistics of China, 2026). Given the absence of a universally accepted single cost-effectiveness threshold in China, 2 times GDP *per capita* was used as the reference-case WTP threshold, corresponding to US$29,399.70/QALY. An ICER below this threshold was considered cost-effective.

### Sensitivity analyses and scenario analyses

To assess model robustness and quantify the impact of parameter uncertainty, one-way sensitivity analysis and probabilistic sensitivity analysis were performed. In the one-way sensitivity analysis, each parameter was varied individually within a prespecified range: utility parameters were varied around the base-case value, and costs and other model parameters were varied over their corresponding uncertainty intervals. The impact on ICER was presented using a tornado diagram. Probabilistic sensitivity analysis was conducted using 5,000 Monte Carlo simulations. Cost parameters were assigned Gamma distributions, while utility and probability parameters were assigned Beta distributions (de Soárez et al., 2014). Results were presented using a cost-effectiveness plane and a cost-effectiveness acceptability curve (CEAC).

This study conducted two scenario analyses to evaluate the robustness of the model results with respect to the duration of long-term treatment effects and permanent discontinuation. Except for the parameters specific to each scenario, all other model assumptions and parameters were kept consistent with those of the base-case analysis.

Scenario 1: Long-term treatment effect duration scenarios. In the base-case analysis, the incremental treatment effect of aficamten versus standard of care (SoC) was assumed to persist through week 96, followed by a linear decline over time and complete disappearance in the third year of treatment. To assess the impact of assumptions regarding the duration of long-term treatment effects on the cost-effectiveness results, three additional scenarios were considered (National Center for Cardiovascular Diseases Cardiomyopathy Alliance, 2025): short-term effect scenario: only the costs and treatment effects of aficamten during the first 24 weeks were considered, with no additional long-term incremental treatment effect retained thereafter (Ommen et al., 2024); 5-year effect scenario: the incremental treatment effect was assumed to persist through week 96, followed by a linear decline and complete disappearance in the fifth year of treatment; and (Li and Wang, 2023) 10-year effect scenario: the incremental treatment effect was assumed to persist through week 96, followed by a linear decline and complete disappearance in the 10th year of treatment. For the 5-year and 10-year effect scenarios, if the incremental cost-effectiveness ratio (ICER) of aficamten versus SoC exceeded the prespecified willingness-to-pay (WTP) threshold, a price threshold analysis was additionally performed to estimate the critical aficamten price required to achieve an acceptable level of cost-effectiveness.

Scenario 2: Permanent discontinuation scenario. Based on data from the FOREST-HCM study, a permanent discontinuation probability of 0.131% per 4-week cycle was incorporated into the model (Tower-Rader et al., 2026). Following permanent discontinuation, aficamten acquisition and aficamten-specific monitoring costs were discontinued from the subsequent cycle onward, and patients continued to receive SoC. The NYHA improvement attributable to aficamten was assumed to be lost after permanent discontinuation, after which patients followed the long-term natural disease progression of the SoC group. Aficamten was not reinitiated after permanent discontinuation. All other model assumptions and parameters remained unchanged from the base-case analysis.

## Results

### Base-case analysis

Base-case results are presented in Table 4. Under the base-case assumption that the incremental treatment effect of aficamten persisted through week 96, followed by a linear waning of effect and complete loss of the incremental benefit by year 3, the total costs and QALYs were US$143,281.22 and 9.696, respectively, for the aficamten plus SoC group, compared with US$96,509.03 and 7.643, respectively, for the SoC-alone group. Compared with SoC alone, aficamten plus SoC was associated with an incremental cost of US$46,772.19 and an incremental gain of 2.054 QALYs, resulting in an ICER of US$22,775.17/QALY, which was below the WTP threshold of US$29,399.70/QALY. Therefore, aficamten plus SoC was cost-effective in the base-case analysis.

**TABLE 4:** Incremental cost-effectiveness results for aficamten plus SoC versus SoC.

Group	Cost (USD)	QALYs	Incremental cost (USD)	Incremental QALYs	ICER (USD/QALY)
Aficamten + SoC	143,281.22	9.696	46,772.19	2.054	22,775.17
SoC	96,509.03	7.643	​	​	​

ICER, incremental cost-effectiveness ratio; QALY, quality-adjusted life-year; SoC,standard of care.

### One-way sensitivity analysis

The results of the one-way sensitivity analysis are shown in Figure 2. The utility value for NYHA class I was the parameter with the greatest impact on the ICER, followed by the acquisition cost of aficamten and the utility value for NYHA class II. A lower utility value for NYHA class I or a higher aficamten drug price resulted in an increased ICER, and at the extreme values of these parameters, the ICER could exceed the WTP threshold.

**FIGURE 2:**
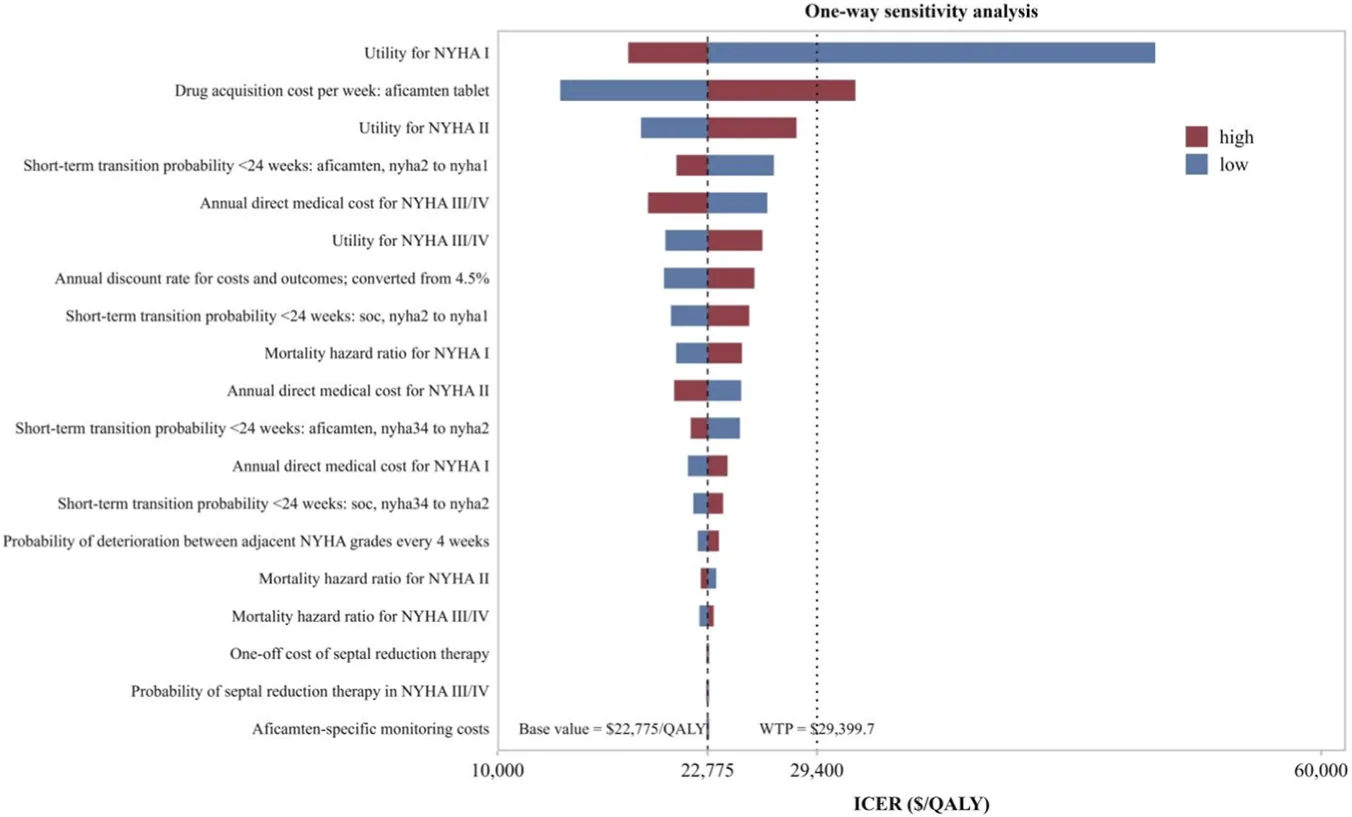
One-way sensitivity analysis tornado diagram for the ICER of aficamten plus SoC versus SoC. Horizontal bars represent the ICER obtained at lower and upper parameter values, blue bars represent lower values, red bars represent upper values, the vertical solid line represents the base-case ICER, the vertical dotted line represents the WTP threshold, and the x-axis represents ICER in USD per QALY.

Other relatively influential parameters included the short-term transition probability from NYHA class II to NYHA class I in the aficamten group during the first 24 weeks, the annual direct medical cost of NYHA class III/IV, the utility value for NYHA class III/IV, and the discount rate. In contrast, the long-term probability of NYHA disease progression, mortality hazard ratios, SRT cost, and probability of undergoing SRT had relatively limited effects on the ICER.

Overall, with the exception of the NYHA class I utility value and aficamten acquisition cost, varying most parameters by ±20% did not alter the cost-effectiveness conclusion of the base-case analysis, suggesting that the model results were generally robust.

### Probabilistic sensitivity analysis

The results of the probabilistic sensitivity analysis are shown in Figures 3, 4. At a WTP threshold of US$29,399.70/QALY, the probabilistic sensitivity analysis showed that most simulations favored aficamten plus SoC as a cost-effective strategy. The cost-effectiveness plane indicated that the majority of simulated points fell below the WTP threshold line, while the CEAC showed a 76.6% probability that aficamten plus SoC was cost-effective, compared with 23.4% for SoC alone. As the WTP threshold increased, the probability that aficamten plus SoC was cost-effective gradually increased and approached 100% at higher WTP thresholds.

**FIGURE 3:**
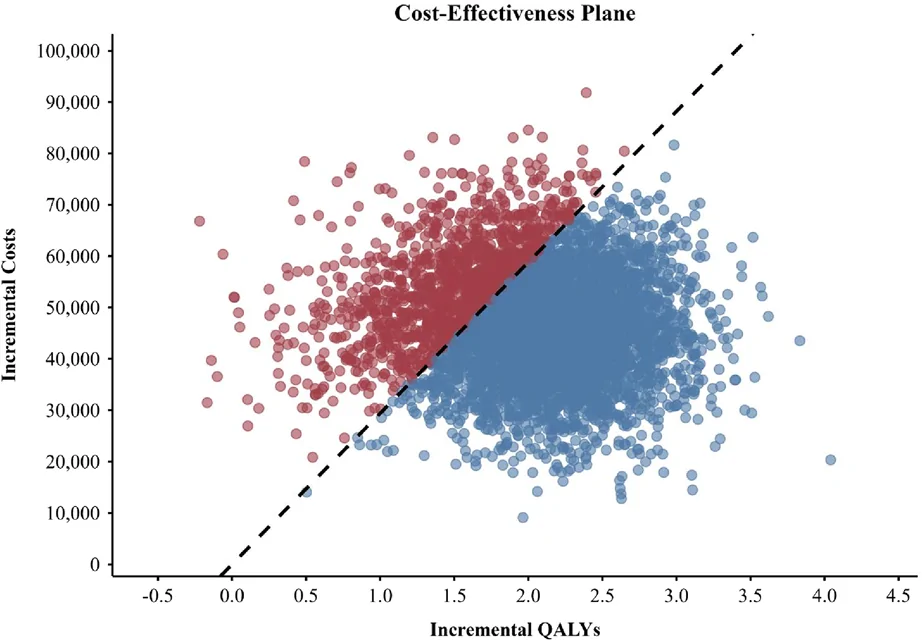
Cost-effectiveness plane from probabilistic sensitivity analysis. Circles represent Monte Carlo simulations, blue circles indicate simulations below the WTP threshold line, red circles indicate simulations above the WTP threshold line, the dashed line represents the WTP threshold, the x-axis represents incremental QALYs, and the y-axis represents incremental costs.

**FIGURE 4:**
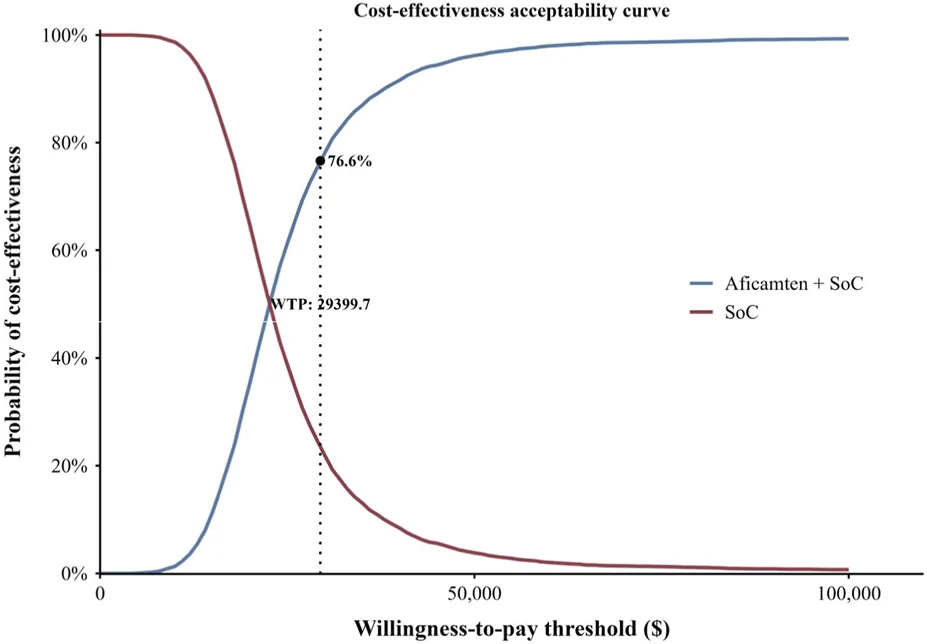
Cost-effectiveness acceptability curve across WTP thresholds. The blue line represents aficamten plus SoC, the red line represents SoC, the vertical dotted line represents the WTP threshold of $29,399.7 per QALY, and the black circle indicates the 76.6% probability that aficamten plus SoC is cost-effective at this threshold.

### Scenario analyses

The scenario analysis results are presented in Table 5. Scenario 1 evaluated the impact of the duration of long-term treatment effects on the cost-effectiveness results. In the short-term scenario, which considered only treatment costs and effects during the first 24 weeks, the incremental cost was USD 16,665.17, the incremental QALY was 0.861, and the ICER was USD 19,351.77/QALY, below the WTP threshold of USD 29,399.70/QALY. When the incremental treatment effect of aficamten completely disappeared in Years 5 and 10, the incremental QALYs were 2.238 and 2.509, respectively, while the incremental costs increased to USD 97,921.60 and USD 203,702.35, resulting in ICERs of USD 43,752.42/QALY and USD 81,220.72/QALY, respectively, both exceeding the WTP threshold. Price threshold analysis showed that the aficamten price would need to be reduced by 22.0% when the treatment effect completely disappeared in Year 5 and by 50.5% when it disappeared in Year 10 to meet the WTP threshold. Overall, as the duration of the treatment effect increased, patients gained greater cumulative health benefits, but the continued accumulation of aficamten drug costs was more pronounced, resulting in progressively higher ICERs.

**TABLE 5:** Scenario analysis results.

Scenario	Incremental cost (USD)	Incremental QALYs	ICER (USD/QALY)	Price reduction required to meet the WTP threshold
Scenario 1. Long-term treatment effect duration				
Short-term costs and effects only	16,665.17	0.861	19,351.77	-
Complete loss of treatment effect by year 5	97,921.60	2.238	43,752.42	22.0%
Complete loss of treatment effect by year 10	203,702.35	2.509	81,220.72	50.5%
Scenario 2. Permanent discontinuation				
Permanent discontinuation of aficamten	46,566.47	1.949	23,888.94	-

ICER, incremental cost-effectiveness ratio; QALY, quality-adjusted life-year; WTP, threshold is USD, 29,399.70 per QALY.

In Scenario 2, incorporating a permanent discontinuation probability of 0.131% per 4-week cycle, the incremental cost was US$46,566.47, the incremental QALY was 1.949, and the ICER was US$23,888.94/QALY, which remained below the WTP threshold of US$29,399.70/QALY. Compared with the base-case analysis, permanent discontinuation reduced the incremental health benefit, while the reduction in aficamten-related treatment costs partially offset the loss of health benefits; nevertheless, the ICER remained below the WTP threshold.

## Discussion

This study developed a four-state Markov model based on the phase III randomized controlled SEQUOIA-HCM trial to evaluate the cost-effectiveness of aficamten plus SoC for patients with symptomatic oHCM from the perspective of the Chinese healthcare system. In the base-case analysis, the incremental treatment effect of aficamten was assumed to persist through week 96, followed by gradual waning and complete loss of the incremental effect by year 3. The results showed that, compared with SoC alone, aficamten plus SoC was associated with an incremental cost of US$46,772.19 and an incremental gain of 2.054 QALYs, yielding an ICER of US$22,775.17/QALY, which was below the prespecified WTP threshold of US$29,399.70/QALY. Therefore, at the current drug price and under the base-case assumptions, aficamten plus SoC was cost-effective. Probabilistic sensitivity analysis showed that, at this WTP threshold, the probability of aficamten plus SoC being cost-effective was 76.6%, further supporting the base-case findings while also indicating that some parameter uncertainty remained in the economic decision.

One-way sensitivity analysis showed that the health utility value for NYHA class I was the most influential parameter affecting the ICER, followed by the acquisition cost of aficamten and the health utility value for NYHA class II. In contrast, the long-term probability of NYHA deterioration, mortality hazard ratios, and SRT-related parameters had relatively limited effects on the ICER. These findings suggest that the economic value of aficamten is primarily determined by two competing factors. On the one hand, improvement in NYHA functional class can increase patients’ health utility and reduce disease-management costs associated with more severe NYHA states. On the other hand, the relatively high acquisition cost of aficamten increases long-term treatment expenditures. Therefore, the quality-of-life gains achieved when patients improve from more symptomatic states to NYHA class I or II, together with the price of aficamten, are key determinants of whether aficamten represents a cost-effective treatment option.

Long-term treatment effect duration is an important source of structural uncertainty in this study. SEQUOIA-HCM primarily provides randomized controlled evidence over 24 weeks, while FOREST-HCM provides further support for the durability of aficamten’s long-term treatment effects. Therefore, rather than assuming that the treatment effect of aficamten would persist indefinitely, we evaluated different scenarios for the duration of long-term treatment effects based on the available clinical evidence. The scenario analysis showed that, in the conservative scenario considering only treatment costs and effects during the first 24 weeks, the ICER was USD 19,351.77/QALY, below the WTP threshold of USD 29,399.70/QALY. When the incremental treatment effect completely disappeared in Years 5 and 10, the incremental QALYs increased to 2.238 and 2.509, respectively, whereas the ICER increased to USD 43,752.42/QALY and USD 81,220.72/QALY, respectively, both exceeding the WTP threshold. Price threshold analysis further showed that, to achieve the WTP threshold, the aficamten price would need to be reduced by 22.0% when the treatment effect completely disappeared in Year 5 and by 50.5% when it completely disappeared in Year 10. These findings indicate that extending the duration of the treatment effect can provide greater cumulative health benefits, but it also implies a longer duration of aficamten treatment, resulting in continued accumulation of drug costs that may exceed the additional health benefits and savings in disease management costs, thereby increasing the ICER. Therefore, the alignment between the duration of treatment benefits and the duration of treatment costs is an important determinant of the long-term cost-effectiveness of aficamten.

Permanent discontinuation is another important source of uncertainty in the long-term cost-effectiveness of aficamten. In Scenario 2, incorporating a permanent discontinuation probability of 0.131% per 4-week cycle reduced the incremental QALY from 2.054 in the base-case analysis to 1.949, while the incremental cost decreased from US$46,772.19 to US$46,566.47. The resulting ICER increased to US$23,888.94/QALY but remained below the WTP threshold of US$29,399.70/QALY. This finding suggests that, despite the loss of treatment-related health benefits following permanent discontinuation, the associated reduction in aficamten drug and monitoring costs partly offset the reduction in QALYs, and the cost-effectiveness conclusion remained robust under the assumed discontinuation rate observed in FOREST-HCM.

This study has several strengths. First, short-term treatment effects were derived directly from the phase III randomized controlled SEQUOIA-HCM trial, and the week-24 NYHA transition matrix was explicitly applied only once, rather than repeatedly at multiple assessment visits during the trial. This approach avoided misinterpreting cumulative improvement proportions as repeated cycle-specific transition probabilities. Second, the long-term model incorporated both treatment-effect waning and permanent treatment discontinuation, allowing uncertainty beyond the randomized controlled trial follow-up period to be handled more realistically. Third, aficamten acquisition costs were weighted according to the dose-titration schedule and the proportions of patients receiving different doses in SEQUOIA-HCM, and aficamten-specific monitoring costs were modeled separately, thereby making the estimation of treatment costs more consistent with the actual treatment pathway. In addition, parameters related to disease-management costs, health-state utilities, mortality risks, and SRT were derived, wherever possible, from Chinese populations or the Chinese healthcare setting. Parameter and structural uncertainty were systematically assessed through one-way sensitivity analysis, probabilistic sensitivity analysis, and long-term treatment-effect scenario analyses.

Several limitations should also be acknowledged. First, publicly available SEQUOIA-HCM data did not provide individual-level transition matrices stratified by baseline NYHA class. Therefore, this study assumed that patients with baseline NYHA class II and NYHA class III/IV had the same probability of improving by at least one NYHA class. Although this assumption was examined in sensitivity analyses, it may not fully capture the true transition patterns of patients with different baseline disease severities. Second, long-term randomized controlled evidence on treatment effects beyond 96 weeks is lacking, and the base-case assumption that the incremental effect gradually wanes and disappears completely by year 3 represents a structural extrapolation. The scenario analyses showed that the duration of long-term treatment effect can substantially influence the ICER; therefore, longer-term controlled studies and real-world evidence will be particularly important for reducing this uncertainty in the future. Third, SRT was simplified in the model as a one-time subsequent treatment event, with the same conditional probability of SRT applied to eligible patients in both groups. Although the model accounted for NYHA improvement following SRT and subsequent disease progression, it did not separately incorporate perioperative mortality, major complications, or the associated utility losses and management costs, which may have led to an underestimation of the overall clinical and economic burden associated with the SRT pathway. Fourth, long-term mortality was extrapolated primarily using age-specific all-cause mortality rates from the general Chinese population adjusted by NYHA-specific mortality hazard ratios. Direct evidence regarding the effect of aficamten on long-term hard clinical endpoints, such as mortality, remains unavailable. In addition, the model did not differentiate background mortality by sex, and therefore may not fully reflect the actual mortality pattern of the study population. Fifth, this analysis was conducted from the perspective of the Chinese healthcare system and included only direct medical costs. Patient time costs, productivity losses, and caregiver burden were not considered; therefore, the findings cannot be directly generalized to a societal perspective. Future studies should further validate the model parameters and predicted outcomes using multicenter real-world data from China.

## Conclusion

This study demonstrates that, at the current price and under the base-case assumptions, aficamten plus SoC is cost-effective compared with SoC alone for the treatment of symptomatic oHCM. However, its cost-effectiveness is sensitive to the drug price, NYHA health-state utility values, and the duration of the long-term treatment effect. Future availability of longer-term clinical and real-world evidence, together with data on actual reimbursement or acquisition prices, will help further reduce decision uncertainty and provide more robust pharmacoeconomic evidence to inform the clinical use and reimbursement decision-making of aficamten.

## Data Availability

The original contributions presented in the study are included in the article/Supplementary Material, further inquiries can be directed to the corresponding author.
