# Supplementary material for: Cost-effectiveness of aficamten for obstructive hypertrophic cardiomyopathy: a model-based analysis from the perspective of the Chinese healthcare system
Source: Front Pharmacol. 2026 Oct 7;17:1938259. doi: 10.3389/fphar.2026.1938259 (PMC13683131; doi:10.3389/fphar.2026.1938259)
Supplement: Supplementary file 1 [file Supplementaryfile1.docx]

**Table S1.** CHEERS 2022 Checklist

**Figure 1.** Markov model structure with NYHA class I, NYHA class II, NYHA class III/IV, and death health states

**Figure 2.** One-way sensitivity analysis tornado diagram for the ICER of aficamten plus SoC versus SoC

**Figure 3.** Cost-effectiveness plane from probabilistic sensitivity analysis

**Figure 4** Cost-effectiveness acceptability curve across WTP thresholds

**Supplementary Data Sheet S1.** R code

**Table S1** CHEERS 2022 Checklist

|  | **Item** | **Guidance for Reporting** | **Is it reported?** |
| --- | --- | --- | --- |
| **Title** | | |  |
| Title | 1 | Identify the study as an economic evaluation and specify the interventions being compared. | √ |
| **Abstract** | | |  |
| Abstract | 2 | Provide a structured summary that highlights context, key methods, results, and alternative analyses. | √ |
| **Introduction** | | |  |
| Introduction: Background and Objectives | 3 | Give the context for the study, the study question, and its practical relevance for decision making in policy or practice. | √ |
| **Methods** | | |  |
| Health economic analysis plan | 4 | Indicate whether a health economic analysis plan was developed and where available. | √ |
| Study population | 5 | Describe characteristics of the study population (such as age range, demographics, socioeconomic, or clinical characteristics). | √ |
| Setting and location | 6 | Provide relevant contextual information that may influence findings. | √ |
| Comparators | 7 | Describe the interventions or strategies being compared and why chosen. | √ |
| Perspective | 8 | State the perspective(s) adopted by the study and why chosen. | √ |
| Time horizon | 9 | State the time horizon for the study and why appropriate. | √ |
| Discount rate | 10 | Report the discount rate(s) and reason chosen. | √ |
| Selection of outcomes | 11 | Describe what outcomes were used as the measure(s) of benefit(s) and harm(s). | √ |
| Measurement of outcomes | 12 | Describe how outcomes used to capture benefit(s) and harm(s) were measured. | √ |
| Valuation of outcomes | 13 | Describe the population and methods used to measure and value outcomes. | √ |
| Measurement and valuation of resources and costs | 14 | Describe how costs were valued. | √ |
| Currency, price date, and conversion | 15 | Report the dates of the estimated resource quantities and unit costs, plus the currency and year of conversion. | √ |
| Rationale and description of model | 16 | If modeling is used, describe in detail and why used. Report if the model is publicly available and where it can be accessed. | √ |
| Analytics and assumptions | 17 | Describe any methods for analyzing or statistically transforming data, any extrapolation methods, and approaches for validating any model used. | √ |
| Characterizing heterogeneity | 18 | Describe any methods used for estimating how the results of the study vary for subgroups. | NA |
| Characterizing distributional effects | 19 | Describe any methods used for estimating how the results of the study vary for subgroups. | NA |
| Characterizing uncertainty | 20 | Describe methods to characterize any sources of uncertainty in the analysis. | √ |
| Approach to engagement with patients and others affected by the study | 21 | Describe any approaches to engage patients or service recipients, the general public, communities, or stakeholders (eg, clinicians or payers) in the design of the study. | × |
| **Results** | | |  |
| Study parameters | 22 | Report all analytic inputs (eg, values, ranges, references) including uncertainty or distributional assumptions. | √ |
| Summary of main results | 23 | Report the mean values for the main categories of costs and outcomes of interest and summarize them in the most appropriate overall measure. | √ |
| Effect of uncertainty | 24 | Describe how uncertainty about analytic judgments, inputs, or projections affects findings. Report the effect of choice of discount rate and time horizon, if applicable. | √ |
| Effect of engagement with patients and others affected by the study | 25 | Report on any difference patient/service recipient, general public, community, or stakeholder involvement made to the approach or findings of the study. | NA |
| **Discussion** | | |  |
| Study findings, limitations, generalizability, and current knowledge | 26 | Report key findings, limitations, ethical, or equity considerations not captured and how these could impact patients, policy, or practice. | √ |
| **Other Relevant Information** | | |  |
| Source of funding | 27 | Describe how the study was funded and any role of the funder in the identification, design, conduct, and reporting of the analysis. | √ |
| Conflicts of interest | 28 | Report authors’ conflicts of interest according to journal or International Committee of Medical Journal Editors requirements. | √ |

**Figure 1** Markov model structure with NYHA class I, NYHA class II, NYHA class III/IV, and death health states

**Figure 2** One-way sensitivity analysis tornado diagram for the ICER of aficamten plus SoC versus SoC. Horizontal bars represent the ICER obtained at lower and upper parameter values, blue bars represent lower values, red bars represent upper values, the vertical solid line represents the base-case ICER, the vertical dotted line represents the WTP threshold, and the x-axis represents ICER in US dollars per QALY.

**Figure 3** Cost-effectiveness plane from probabilistic sensitivity analysis. Circles represent Monte Carlo simulations, blue circles indicate simulations below the WTP threshold line, red circles indicate simulations above the WTP threshold line, the dashed line represents the WTP threshold, the x-axis represents incremental QALYs, and the y-axis represents incremental costs.

**Figure 4** Cost-effectiveness acceptability curve across WTP thresholds. The blue line represents aficamten plus SoC, the red line represents SoC, the vertical dotted line represents the WTP threshold of $29,399.7 per QALY, and the black circle indicates the 76.6% probability that aficamten plus SoC is cost-effective at this threshold.

**Supplementary Data Sheet S1.** R Code for the Pharmacoeconomic Model and Sensitivity Analyses

The complete code can be found at: <https://github.com/fjmuycc-jpg/Afkaitai.git>

#install.packages("rgho")

library(writexl)

library(carcass)

library(survminer)

library(ggplot2)

library(dplyr)

library(heemod)

library(openxlsx)

library(cowplot)

library(gridExtra)

library(ggplot2)

library(scales)

if (!require(ragg)) install.packages("ragg", repos = "https://cloud.r-project.org"); library(ragg)

windowsFonts(

Times = windowsFont("Times New Roman")

)

#library(extrafont)

\# Read parameters

setwd("PATH/TO/PROJECT")

par <- read.xlsx("model_parameters.xlsx")

par1 <- par[,c("par","4-Week Cycle-Adjusted Probability")]

par1 <- par1 %>%

rename(base = `4-Week Cycle-Adjusted Probability`)

par1$par

\# Specify the column containing parameter values

value_col <- "base"

\# Ensure that parameter values are numeric

par1[[value_col]] <- as.numeric(par1[[value_col]])

\# Create a variable for each row in par and assign its value

param_list <- setNames(as.list(par1[[value_col]]), par1$par)

list2env(param_list, envir = .GlobalEnv)

rm(list = par1$par, envir = .GlobalEnv)

p_d <- read.xlsx("p_d.xlsx")

\# 1. Define transition matrices

mat_TG <- define_transition(

state_names = c("nyha1", "nyha2_on", "nyha2_off", "nyha34", "death"),

(1-p_d1)*p_af_nyha1_to_nyha1, 0,

(1-p_d1)*p_af_nyha1_to_nyha2,

(1-p_d1)*p_af_nyha1_to_nyha34, p_d1,

(1-p_d2)*p_af_nyha2_to_nyha1,

(1-p_d2)*p_af_nyha2_to_nyha2, 0,

(1-p_d2)*p_af_nyha2_to_nyha34, p_d2,

(1-p_d2)*p_af_nyha2_to_nyha1, 0,

(1-p_d2)*p_af_nyha2_to_nyha2,

(1-p_d2)*p_af_nyha2_to_nyha34, p_d2,

(1-p_d34)*p_af_nyha34_to_nyha1,

(1-p_d34)*p_af_nyha34_to_nyha2, 0,

(1-p_d34)*p_af_nyha34_to_nyha34, p_d34, 0, 0, 0, 0, 1)

mat_CG <- define_transition(

state_names = c("nyha1", "nyha2_on", "nyha2_off", "nyha34", "death"),

(1-p_d1)*p_cg_nyha1_to_nyha1, 0,

(1-p_d1)*p_cg_nyha1_to_nyha2,

(1-p_d1)*p_cg_nyha1_to_nyha34, p_d1,

(1-p_d2)*p_cg_nyha2_to_nyha1,

(1-p_d2)*p_cg_nyha2_to_nyha2, 0,

(1-p_d2)*p_cg_nyha2_to_nyha34, p_d2,

(1-p_d2)*p_cg_nyha2_to_nyha1, 0,

(1-p_d2)*p_cg_nyha2_to_nyha2,

(1-p_d2)*p_cg_nyha2_to_nyha34, p_d2,

(1-p_d34)*p_cg_nyha34_to_nyha1,

(1-p_d34)*p_cg_nyha34_to_nyha2, 0,

(1-p_d34)*p_cg_nyha34_to_nyha34, p_d34, 0, 0, 0, 0, 1)

\# 2. Define health states

state_nyha1 <- define_state(

cost_drug = discount(dispatch_strategy(

TG = c_soc_week * cycle_length + c_aficamten_cycle,

CG = c_soc_week * cycle_length), r = r_discount_cycle),

cost_medical = discount(c_hcru_nyha1_annual/(52/cycle_length), r = r_discount_cycle),

cost_ae = discount(p_ae_af*c_ae_af_event, r = r_discount_cycle),

cost_total = cost_drug + cost_medical + cost_ae,

utility = discount(dispatch_strategy(

TG = (u_nyha1-ifelse(model_time <= cycle_3y, u_ae_grade34_af*p_ae_af, 0))/(52/cycle_length),

CG = u_nyha1/(52/cycle_length)), r = r_discount_cycle))

state_nyha2_on <- define_state(

cost_drug = discount(dispatch_strategy(

TG = c_soc_week * cycle_length + c_aficamten_cycle,

CG = c_soc_week * cycle_length), r = r_discount_cycle),

cost_medical = discount(c_hcru_nyha2_annual/(52/cycle_length), r = r_discount_cycle),

cost_ae = discount(p_ae_af*c_ae_af_event, r = r_discount_cycle),

cost_total = cost_drug + cost_medical + cost_ae,

utility = discount(dispatch_strategy(

TG = (u_nyha2-ifelse(model_time <= cycle_3y, u_ae_grade34_af*p_ae_af, 0))/(52/cycle_length),

CG = u_nyha2/(52/cycle_length)), r = r_discount_cycle))

state_nyha2_off <- define_state(

cost_drug = discount(dispatch_strategy(

TG = c_soc_week * cycle_length + c_aficamten_cycle,

CG = c_soc_week * cycle_length), r = r_discount_cycle),

cost_medical = discount(c_hcru_nyha2_annual/(52/cycle_length), r = r_discount_cycle),

cost_ae = discount(ifelse(model_time <= cycle_3y, p_ae_af * c_ae_af_event, 0),r = r_discount_cycle),cost_total = cost_drug + cost_medical + cost_ae,

utility = discount(dispatch_strategy(

TG = (u_nyha2-ifelse(model_time <= cycle_3y, u_ae_grade34_af*p_ae_af, 0))/(52/cycle_length),

CG = u_nyha2/(52/cycle_length)), r = r_discount_cycle))

state_nyha34 <- define_state( cost_drug = discount(dispatch_strategy(

TG = c_soc_week * cycle_length + c_aficamten_cycle,

CG = c_soc_week * cycle_length), r = r_discount_cycle),

cost_medical = discount(

c_hcru_nyha34_annual/(52/cycle_length) + p_srt_nyha34*c_srt_event,

r = r_discount_cycle),

cost_ae = discount(p_ae_af*c_ae_af_event, r = r_discount_cycle),

cost_total = cost_drug + cost_medical + cost_ae,

utility = discount(dispatch_strategy(

TG = (u_nyha34-ifelse(model_time <= cycle_3y, u_ae_grade34_af*p_ae_af, 0))/(52/cycle_length),

CG = u_nyha34/(52/cycle_length)), r = r_discount_cycle))

state_d <- define_state(

cost_drug = 0,

cost_medical = 0,

cost_ae = 0,

cost_total = 0,

utility = 0)

\# 3. Define strategies

strat_TG <- define_strategy(

transition = mat_TG,

nyha1 = state_nyha1,

nyha2_on = state_nyha2_on,

nyha2_off = state_nyha2_off,

nyha34 = state_nyha34,

death = state_d)

strat_CG <- define_strategy(

transition = mat_CG,

nyha1 = state_nyha1,

nyha2_on = state_nyha2_on,

nyha2_off = state_nyha2_off,

nyha34 = state_nyha34,

death = state_d)

\# 4. Parameters

parameters <- define_parameters(

\# Basic parameters

age_start = par1$base[par1$par == "age_start"],

short_term_weeks = par1$base[par1$par == "short_term_weeks"],

cycle_length = par1$base[par1$par == "cycle_length"],

cycle1 = short_term_weeks/cycle_length,

\# Long-term treatment effect: incremental effect maintained through week 96, followed by waning, with complete loss by year 5 cycle_96w = 96/cycle_length,

cycle_3y = 3*52/cycle_length,

af_effect = ifelse( model_time <= cycle_96w, 1,

ifelse( model_time <= cycle_3y,

(cycle_3y-model_time)/(cycle_3y-cycle_96w), 0 ) ),

\# Discounting

r_discount = par1$base[par1$par == "r_discount"],

r_discount_cycle = (1+r_discount)^(cycle_length/52)-1,

gdp_per_capita = par1$base[par1$par == "gdp_per_capita"],

wtp_threshold = par1$base[par1$par == "wtp_threshold"],

n_psa = par1$base[par1$par == "n_psa"],

\# Initial health-state distribution

p_init_nyha1 = par1$base[par1$par == "p_init_nyha1"],

p_init_nyha2 = par1$base[par1$par == "p_init_nyha2"],

p_init_nyha34 = par1$base[par1$par == "p_init_nyha34"],

\# Aficamten short-term transition probabilities: define independent probabilities only p_st_aficamten_nyha1_to_nyha2 = 0,

p_st_aficamten_nyha1_to_nyha34 = 0,

p_st_aficamten_nyha1_to_nyha1 =

1-p_st_aficamten_nyha1_to_nyha2-p_st_aficamten_nyha1_to_nyha34,

p_st_aficamten_nyha2_to_nyha1 =

par1$base[par1$par == "p_st_aficamten_nyha2_to_nyha1"],

p_st_aficamten_nyha2_to_nyha34 = 0,

p_st_aficamten_nyha2_to_nyha2 =

1-p_st_aficamten_nyha2_to_nyha1-p_st_aficamten_nyha2_to_nyha34,

p_st_aficamten_nyha34_to_nyha1 = 0,

p_st_aficamten_nyha34_to_nyha2 =

par1$base[par1$par == "p_st_aficamten_nyha34_to_nyha2"],

p_st_aficamten_nyha34_to_nyha34 =

1-p_st_aficamten_nyha34_to_nyha1-p_st_aficamten_nyha34_to_nyha2,

\# SoC short-term transition probabilities: define independent probabilities only p_st_soc_nyha1_to_nyha2 = 0,

p_st_soc_nyha1_to_nyha34 = 0,

p_st_soc_nyha1_to_nyha1 =

1-p_st_soc_nyha1_to_nyha2-p_st_soc_nyha1_to_nyha34,

p_st_soc_nyha2_to_nyha1 =

par1$base[par1$par == "p_st_soc_nyha2_to_nyha1"],

p_st_soc_nyha2_to_nyha34 = 0,

p_st_soc_nyha2_to_nyha2 =

1-p_st_soc_nyha2_to_nyha1-p_st_soc_nyha2_to_nyha34,

p_st_soc_nyha34_to_nyha1 = 0,

p_st_soc_nyha34_to_nyha2 =

par1$base[par1$par == "p_st_soc_nyha34_to_nyha2"],

p_st_soc_nyha34_to_nyha34 =

1-p_st_soc_nyha34_to_nyha1-p_st_soc_nyha34_to_nyha2,

\# Long-term natural progression probabilities

p_deteriorate = par1$base[par1$par == "p_deteriorate"],

p_lt_nyha1_to_nyha2 = p_deteriorate,

p_lt_nyha1_to_nyha34 = 0,

p_lt_nyha1_to_nyha1 =1-p_lt_nyha1_to_nyha2-p_lt_nyha1_to_nyha34,

p_lt_nyha2_to_nyha1 = 0,

p_lt_nyha2_to_nyha34 = p_deteriorate,

p_lt_nyha2_to_nyha2 =

1-p_lt_nyha2_to_nyha1-p_lt_nyha2_to_nyha34,

p_lt_nyha34_to_nyha1 = 0,

p_lt_nyha34_to_nyha2 = 0,

p_lt_nyha34_to_nyha34 =

1-p_lt_nyha34_to_nyha1-p_lt_nyha34_to_nyha2,

\# Incremental transition effects of aficamten relative to SoC observed in the RCT te_nyha1_to_nyha2 =

p_st_aficamten_nyha1_to_nyha2-p_st_soc_nyha1_to_nyha2,

te_nyha1_to_nyha34 =

p_st_aficamten_nyha1_to_nyha34-p_st_soc_nyha1_to_nyha34,

te_nyha2_to_nyha1 =

p_st_aficamten_nyha2_to_nyha1-p_st_soc_nyha2_to_nyha1,

te_nyha2_to_nyha34 =

p_st_aficamten_nyha2_to_nyha34-p_st_soc_nyha2_to_nyha34,

te_nyha34_to_nyha1 =

p_st_aficamten_nyha34_to_nyha1-p_st_soc_nyha34_to_nyha1,

te_nyha34_to_nyha2 =

p_st_aficamten_nyha34_to_nyha2-p_st_soc_nyha34_to_nyha2,

\# CG dynamic transition probabilities: use SoC data during the trial phase, then long-term natural progression

p_cg_nyha1_to_nyha2 =

ifelse(model_time <= cycle1, p_st_soc_nyha1_to_nyha2, p_lt_nyha1_to_nyha2),

p_cg_nyha1_to_nyha34 =

ifelse(model_time <= cycle1, p_st_soc_nyha1_to_nyha34, p_lt_nyha1_to_nyha34),

p_cg_nyha1_to_nyha1 = 1-p_cg_nyha1_to_nyha2-p_cg_nyha1_to_nyha34,

p_cg_nyha2_to_nyha1 =

ifelse(model_time <= cycle1, p_st_soc_nyha2_to_nyha1, p_lt_nyha2_to_nyha1),

p_cg_nyha2_to_nyha34 =

ifelse(model_time <= cycle1, p_st_soc_nyha2_to_nyha34, p_lt_nyha2_to_nyha34),

p_cg_nyha2_to_nyha2 = 1-p_cg_nyha2_to_nyha1-p_cg_nyha2_to_nyha34,

p_cg_nyha34_to_nyha1 =

ifelse(model_time <= cycle1, p_st_soc_nyha34_to_nyha1, p_lt_nyha34_to_nyha1),

p_cg_nyha34_to_nyha2 =

ifelse(model_time <= cycle1, p_st_soc_nyha34_to_nyha2, p_lt_nyha34_to_nyha2),

p_cg_nyha34_to_nyha34 = 1-p_cg_nyha34_to_nyha1-p_cg_nyha34_to_nyha2,

\# TG dynamic transition probabilities: use aficamten data during the trial phase; thereafter = natural progression + remaining incremental treatment effect

p_af_nyha1_to_nyha2 = ifelse(

model_time <= cycle1,

p_st_aficamten_nyha1_to_nyha2,

p_lt_nyha1_to_nyha2 + af_effect*te_nyha1_to_nyha2

),

p_af_nyha1_to_nyha34 = ifelse(

model_time <= cycle1,

p_st_aficamten_nyha1_to_nyha34,

p_lt_nyha1_to_nyha34 + af_effect*te_nyha1_to_nyha34

),

p_af_nyha1_to_nyha1 = 1-p_af_nyha1_to_nyha2-p_af_nyha1_to_nyha34,

p_af_nyha2_to_nyha1 = ifelse(

model_time <= cycle1,

p_st_aficamten_nyha2_to_nyha1,

p_lt_nyha2_to_nyha1 + af_effect*te_nyha2_to_nyha1

),

p_af_nyha2_to_nyha34 = ifelse(

model_time <= cycle1,

p_st_aficamten_nyha2_to_nyha34,

p_lt_nyha2_to_nyha34 + af_effect*te_nyha2_to_nyha34

),

p_af_nyha2_to_nyha2 = 1-p_af_nyha2_to_nyha1-p_af_nyha2_to_nyha34,

p_af_nyha34_to_nyha1 = ifelse(

model_time <= cycle1,

p_st_aficamten_nyha34_to_nyha1,

p_lt_nyha34_to_nyha1 + af_effect*te_nyha34_to_nyha1

),

p_af_nyha34_to_nyha2 = ifelse(

model_time <= cycle1,

p_st_aficamten_nyha34_to_nyha2,

p_lt_nyha34_to_nyha2 + af_effect*te_nyha34_to_nyha2

),

p_af_nyha34_to_nyha34 = 1-p_af_nyha34_to_nyha1-p_af_nyha34_to_nyha2,

\# Mortality

p_mort_55_59 = par1$base[par1$par == "p_mort_55_59"],

p_mort_60_64 = par1$base[par1$par == "p_mort_60_64"],

p_mort_65_69 = par1$base[par1$par == "p_mort_65_69"],

p_mort_70_74 = par1$base[par1$par == "p_mort_70_74"],

p_mort_75_79 = par1$base[par1$par == "p_mort_75_79"],

p_mort_80_84 = par1$base[par1$par == "p_mort_80_84"],

p_mort_85_89 = par1$base[par1$par == "p_mort_85_89"],

p_mort_90_plus = par1$base[par1$par == "p_mort_90_plus"],

hr_mort_nyha1 = par1$base[par1$par == "hr_mort_nyha1"],

hr_mort_nyha2 = par1$base[par1$par == "hr_mort_nyha2"],

hr_mort_nyha34 = par1$base[par1$par == "hr_mort_nyha34"],

p_d1 = 1-(1-p_d[model_time,"p"])^(hr_mort_nyha1*cycle_length/52),

p_d2 = 1-(1-p_d[model_time,"p"])^(hr_mort_nyha2*cycle_length/52),

p_d34 = 1-(1-p_d[model_time,"p"])^(hr_mort_nyha34*cycle_length/52),

\# AEs, SRT, and utilities

p_ae_af = par1$base[par1$par == "p_ae_af"],

p_srt_nyha34 = par1$base[par1$par == "p_srt_nyha34"],

u_nyha1 = par1$base[par1$par == "u_nyha1"],

u_nyha2 = par1$base[par1$par == "u_nyha2"],

u_nyha34 = par1$base[par1$par == "u_nyha34"],

u_ae_grade34_af = par1$base[par1$par == "u_ae_grade34_af"],

\# Costs: aficamten treatment continued through year 5

c_monitoring = par1$base[par1$par == "c_monitoring"],

c_aficamten_week = par1$base[par1$par == "c_aficamten_week"],

c_aficamten_cycle = ifelse(

model_time <= 1,

c_aficamten_week*2.964286 + c_monitoring*2,

ifelse(

model_time <= 2,

c_aficamten_week*6.085714 + c_monitoring*2,

ifelse(

model_time <= 6,

c_aficamten_week*12.928 + c_monitoring*2,

ifelse(

model_time <= cycle_3y,

c_aficamten_week*12.928 + c_monitoring/3.25,0)	)) ),

c_metoprolol_week = par1$base[par1$par == "c_metoprolol_week"],

c_verapamil_week = par1$base[par1$par == "c_verapamil_week"],

p_metoprolol_use = par1$base[par1$par == "p_metoprolol_use"],

c_soc_week = c_metoprolol_week*p_metoprolol_use+c_verapamil_week*(1-p_metoprolol_use),

c_hcru_nyha1_annual = par1$base[par1$par == "c_hcru_nyha1_annual"],

c_hcru_nyha2_annual = par1$base[par1$par == "c_hcru_nyha2_annual"],

c_hcru_nyha34_annual = par1$base[par1$par == "c_hcru_nyha34_annual"],

c_srt_event = par1$base[par1$par == "c_srt_event"],

c_ae_af_event = par1$base[par1$par == "c_ae_af_event"])

\# 5. Run model

res_mod2 <- run_model(

TG = strat_TG,

CG = strat_CG,

parameters = parameters,

cycles = 532,

cost = cost_total,

effect = utility,

method = "life-table",

init = c(0, 0, 0.759, 0.241, 0))

summary(res_mod2)

for (x in seq) {}

parameters_new <- heemod::modify(

parameters,

c_aficamten_week = par1$base[par1$par == "c_aficamten_week"]*x)

res_c_aficamten_cycle <- run_model(

TG = strat_TG,

CG = strat_CG,

parameters = parameters_new,

cycles = 532,

cost = cost_total,

effect = utility,

method = "life-table",

init = c(0, 0, 0.759, 0.241, 0))

29399.70

summary(res_c_aficamten_cycle)

base_c_aficamten <- par1$base[

par1$par == "c_aficamten_week"]

x_seq <- seq(0, 1, by = 0.001)

result_df <- data.frame(

x = x_seq, ICER = NA_real_)

for (i in seq_along(x_seq)) { x <- x_seq[i]

print(i)

parameters_new <- heemod::modify(parameters, c_aficamten_week = base_c_aficamten * x )

res_temp <- run_model(TG = strat_TG, CG = strat_CG,

parameters = parameters_new,

cycles = 532,

cost = cost_total,

effect = utility,

method = "life-table",

init = c(0, 0, 0.759, 0.241, 0) )

a2 <- summary(res_temp)

a2[["res_values"]]$ICER <- (a2[["res_values"]][["cost_total"]][1]-a2[["res_values"]][["cost_total"]][2])/

(a2[["res_values"]][["utility"]][1]-a2[["res_values"]][["utility"]][2])

result_df$ICER[i] <- a2[["res_values"]]$ICER}

head(result_df)

\# Retain base-case analysis results

a2 <- summary(res_mod2)

a2[["res_values"]]$ICER <- (a2[["res_values"]][["cost_total"]][1]-a2[["res_values"]][["cost_total"]][2])/

(a2[["res_values"]][["utility"]][1]-a2[["res_values"]][["utility"]][2])

write.xlsx(a2[["res_values"]], file="results/analysis_results_waning_after_week96_complete_loss_year3.xlsx")

human1 <- get_counts(res_mod2)

head(human1)

cost_df2 <- get_values(res_mod2)

write.xlsx(human, file="human.xlsx")

write.xlsx(cost_df, file="cost_df.xlsx")

human_plot <- human1 %>%

mutate( time_year = model_time * 4 / 52)

ggplot(human_plot, aes(x = time_year, y = count, color = state_names)) +

geom_line(linewidth = 1) +

facet_wrap(~ .strategy_names) +

labs(x = "Time (years)",

y = "Proportion of cohort",

color = "Health state",

title = "State occupancy over time" ) +theme_bw()

\# Sensitivity analyses ##############

setwd("PATH/TO/PROJECT")

par <- read.xlsx("model_parameters.xlsx")

names(par)

par2 <- par[,c("par","4-Week Cycle-Adjusted Probability","low","hight","distribution","a","b","sd")]

names(par2)[names(par2) == "4-Week Cycle-Adjusted Probability"] <- "base"

head(par2)

library(dplyr)

par2 <- par2 %>%

mutate(base = as.numeric(base),

low = as.numeric(low),

hight = as.numeric(hight),

\# Derive the standard error from the lower and upper bounds, assuming low and hight represent a 95% CI or a sensitivity-analysis range

se = (hight - low) / (2 * 1.96),

\# Intermediate term for the Beta distribution

beta_tmp = ifelse(

distribution == "Beta" & base > 0 & base < 1 & se > 0,

base * (1 - base) / se^2 - 1, NA ),

a = case_when(

distribution == "Beta" & !is.na(beta_tmp) & beta_tmp > 0 ~ base * beta_tmp,

distribution == "Gamma" & base > 0 & se > 0 ~ base^2 / se^2,

distribution == "Log-normal" & base > 0 ~ log(base),

TRUE ~ NA_real_ ),

b = case_when(

distribution == "Beta" & !is.na(beta_tmp) & beta_tmp > 0 ~ (1 - base) * beta_tmp,

\# Here b is specified as the scale parameter

distribution == "Gamma" & base > 0 & se > 0 ~ se^2 / base,

TRUE ~ NA_real_ ),

sd = case_when(distribution == "Log-normal" & low > 0 & hight > 0 ~

(log(hight) - log(low)) / (2 * 1.96),

TRUE ~ NA_real_ ) ) %>%

select(-beta_tmp)

par2$distribution <- trimws(tolower(par2$distribution))

par2$dis <- apply(par2, 1, function(row) {

paste0("D_",row["par2"])})

par2 <- par2[!is.na(par2$a), ]

\## Generate distribution-definition code

par2_sentences_gamma <- apply(

par2[par2$distribution == "gamma", ], 1,

function(row) {

paste0(

row["par"], " ~ gamma(",

"mean = ", row["base"], ", ",

"sd = ", row["se"], "),")})

par2_sentences_beta <- apply(

par2[par2$distribution == "beta", ], 1,

function(row) { paste0( row["par"], " ~ beta(",

"shape1 = ", row["a"], ", ",

"shape2 = ", row["b"], "),"	) })

par2_sentences_lognormal <- apply(

par2[par2$distribution %in% c("log-normal", "lognormal", "log normal"), ], 1,

function(row) { paste0( row["par"], " ~ lognormal(",

"meanlog = ", row["a"], ", ",

"sdlog = ", row["sd"], "),")})

par2_sentences_all <- c(

par2_sentences_gamma,

par2_sentences_beta,

par2_sentences_lognormal)

\# Display output

cat(par2_sentences_all, sep = "\n")

sp <- define_psa(

c_aficamten_week ~ gamma(mean = 2.064897e+02, sd = 2.107038e+01),

c_metoprolol_week ~ gamma(mean = 2.333333e-01, sd = 2.377942e-02),

c_verapamil_week ~ gamma(mean = 6.039823e+00, sd = 6.163085e-01),

c_hcru_nyha1_annual ~ gamma(mean = 2.013937e+03, sd = 2.055039e+02),

c_hcru_nyha2_annual ~ gamma(mean = 7.997016e+03, sd = 8.160218e+02),

c_hcru_nyha34_annual ~ gamma(mean = 1.991660e+04, sd = 2.032306e+03),

c_srt_event ~ gamma(mean = 9.912743e+02, sd = 1.011506e+02),

c_ae_af_event ~ gamma(mean = 6.805678e+03, sd = 6.944570e+02),

c_monitoring ~ gamma(mean = 3.834808e+01, sd = 3.913070e+00),

r_discount ~ beta(shape1 = 91.6732000, shape2 = 1.945509e+03),

p_init_nyha2 ~ beta(shape1 = 22.3866400, shape2 = 7.108274e+00),

p_init_nyha34 ~ beta(shape1 = 72.6533600, shape2 = 2.288129e+02),

#p_st_aficamten_nyha2_to_nyha1 ~ beta(shape1 = 82.8092753, shape2 = 5.245507e+02),

#p_st_aficamten_nyha2_to_nyha2 ~ beta(shape1 = 15.4955220, shape2 = 2.446233e+00),

#p_st_aficamten_nyha34_to_nyha2 ~ beta(shape1 = 82.8092753, shape2 = 5.245507e+02),

#p_st_aficamten_nyha34_to_nyha34 ~ beta(shape1 = 15.4955220, shape2 = 2.446233e+00),

#p_st_soc_nyha2_to_nyha1 ~ beta(shape1 = 91.6403034, shape2 = 1.929584e+03),

#p_st_soc_nyha2_to_nyha2 ~ beta(shape1 = 10.4195390, shape2 = 4.948474e-01),

#p_st_soc_nyha34_to_nyha2 ~ beta(shape1 = 91.6403034, shape2 = 1.929584e+03),

#p_st_soc_nyha34_to_nyha34 ~ beta(shape1 = 10.4195390, shape2 = 4.948474e-01),

p_deteriorate ~ beta(shape1 = 95.7100640, shape2 = 2.805431e+04),

p_improvement ~ beta(shape1 = 39.2716000, shape2 = 2.785934e+01),

#p_lt_nyha1_to_nyha1 ~ beta(shape1 = 0.2660984, shape2 = 9.078211e-04),

#p_lt_nyha1_to_nyha2 ~ beta(shape1 = 95.7100640, shape2 = 2.805431e+04),

#p_lt_nyha2_to_nyha2 ~ beta(shape1 = 0.2660984, shape2 = 9.078211e-04),

#p_lt_nyha2_to_nyha34 ~ beta(shape1 = 95.7100640, shape2 = 2.805431e+04),

p_mort_55_59 ~ beta(shape1 = 95.3024960, shape2 = 1.244450e+04),

p_mort_60_64 ~ beta(shape1 = 94.7561608, shape2 = 7.067463e+03),

p_mort_65_69 ~ beta(shape1 = 93.9148240, shape2 = 4.194433e+03),

p_mort_70_74 ~ beta(shape1 = 91.4830016, shape2 = 1.856622e+03),

p_mort_75_79 ~ beta(shape1 = 87.2462352, shape2 = 8.755240e+02),

p_mort_80_84 ~ beta(shape1 = 79.5441704, shape2 = 3.883902e+02),

p_mort_85_89 ~ beta(shape1 = 68.0274632, shape2 = 1.676308e+02),

p_mort_90_plus ~ beta(shape1 = 51.8557472, shape2 = 6.203283e+01),

p_ae_af ~ beta(shape1 = 95.9264632, shape2 = 8.189250e+04),

p_srt_nyha34 ~ beta(shape1 = 92.7651911, shape2 = 2.656077e+03),

u_nyha1 ~ beta(shape1 = 14.5695959, shape2 = 1.440949e+00),

u_nyha2 ~ beta(shape1 = 15.4130234, shape2 = 2.719945e+00),

u_nyha34 ~ beta(shape1 = 28.1120000, shape2 = 1.204800e+01),

u_ae_grade34_af ~ beta(shape1 = 77.6024000, shape2 = 3.308313e+02),

hr_mort_nyha1 ~ lognormal(meanlog = 0.0000000, sdlog = 0.103435),

hr_mort_nyha2 ~ lognormal(meanlog = 0.5877867, sdlog = 0.103435),

hr_mort_nyha34 ~ lognormal(meanlog = 1.5475625, sdlog = 0.103435),

\# TG short term: NYHA II row

p_st_aficamten_nyha2_to_nyha1 +

p_st_aficamten_nyha2_to_nyha2 ~ multinomial(136, 864),

\# TG short term: NYHA III/IV row

p_st_aficamten_nyha34_to_nyha2 +

p_st_aficamten_nyha34_to_nyha34 ~ multinomial(136, 864),

\# CG short term: NYHA II row

p_st_soc_nyha2_to_nyha1 +

p_st_soc_nyha2_to_nyha2 ~ multinomial(45, 955),

\# CG short term: NYHA III/IV row

p_st_soc_nyha34_to_nyha2 +

p_st_soc_nyha34_to_nyha34 ~ multinomial(45, 955),

\# Long term: NYHA I row

p_lt_nyha1_to_nyha1 +

p_lt_nyha1_to_nyha2 ~ multinomial(9966, 34),

\# Long term: NYHA II row

p_lt_nyha2_to_nyha2 +

p_lt_nyha2_to_nyha34 ~ multinomial(9966, 34))

summary(res_mod2)

\## Run probabilistic sensitivity analysis

set.seed(16875)

ndt1 <- run_psa(res_mod2, psa = rsp, N = 5000)

library(dplyr)

library(tidyr)

tab2 <- ndt1$psa %>%

select(.index, .strategy_names, .cost, .effect) %>%

pivot_wider(

names_from = .strategy_names,

values_from = c(.cost, .effect)) %>%

mutate(

Incremental_effect = .effect_TG - .effect_CG,

Incremental_cost = .cost_TG - .cost_CG,

ICER = Incremental_cost / Incremental_effect )

mean(tab2$ICER)

mean(tab2$Incremental_cost) / mean(tab2$Incremental_effect)

write_xlsx(tab2, path = "results/ce_plane_data_waning_after_week96_complete_loss_year5.xlsx")

remove(ndt1)

options(scipen = 999)

\## Plot cost-effectiveness plane

source("plotting_functions.R")

ce_plane_plot <- plot_ce_plane(

df = tab2,

WTP = 29399.70,

scatterplot_name_title = "Cost-Effectiveness Plane",

scatterplot_name_x = "Incremental QALYs",

scatterplot_name_y = "Incremental Costs",

plot_title_size = 14,

x_title_size = 12,

y_title_size = 12,

x_text_size = 12,

y_text_size = 12,

legend_title_size = 12,

legend_text_size = 12,

scatterplot_hjust = 0.5,

color_below = "#4E79A7",

color_above = "#A23E48")

ggsave(

"ce_plane_waning_after_week96_complete_loss_year3.JPG",

plot = ce_plane_plot,

device = ragg::agg_jpeg,

units = "cm",

width = 20,

height = 14,

dpi = 600)

\## Plot cost-effectiveness acceptability curve

source("plotting_functions.R")

ceac_dat <- tab2

ceac_plot <- plot_ceac(

ceac_dat = ceac_dat,

WTP = 29399.70,

ceac_name_title = "Cost-effectiveness acceptability curve",

ceac_name_x = "Willingness-to-pay threshold ($)",

ceac_name_y = "Probability of cost-effectiveness",

ceac_hjust = 0.5,

limit_x = 100000,

by_x = 50000,

xx = 1.1,

by_scatter = 1000,

ceac_label_TG = "Aficamten + SoC",

ceac_label_CG = "SoC",

color_TG = "#4E79A7",

color_CG = "#A23E48"

)

mean(tab2$ICER)

ggsave("ceac_waning_after_week96_complete_loss_year3.JPG", plot = ceac_plot, units = "cm", width = 20, height = 14, dpi = 600)

\## One-way sensitivity analysis

source("plotting_functions.R")

options(scipen = 999)

a1 <- apply(par2, 1, function(row) {

paste0(row["par"], ", ", row["low"], ",",row["hight"],",")

})cat(a1, sep = "\n")

a1_df <- data.frame(Formula = unlist(a1))

write.xlsx(a1_df,file = "a1_df.xlsx")

summary(res_mod2)

dsa <- define_dsa( #### Discount rate ####

r_discount, 0.036, 0.054,

\#### Short-term treatment effect: include improvement probabilities only; exclude stay probabilities ####

p_st_aficamten_nyha2_to_nyha1, 0.1090744, 0.1636116,

p_st_aficamten_nyha34_to_nyha2, 0.1090744, 0.1636116,

p_st_soc_nyha2_to_nyha1, 0.0362712, 0.0544068,

p_st_soc_nyha34_to_nyha2, 0.0362712, 0.0544068,

\#### Long-term deterioration probability: include p_deteriorate only ####

p_deteriorate, 0.0027200, 0.0040800,

\#### Week-24 improvement rate / treatment-continuation-related parameter ####

#p_improvement, 0.4680000, 0.7020000,

\#### Mortality ####

p_mort_55_59, 0.0060800, 0.0091200,

p_mort_60_64, 0.0105840, 0.0158760,

p_mort_65_69, 0.0175200, 0.0262800,

p_mort_70_74, 0.0375680, 0.0563520,

p_mort_75_79, 0.0724960, 0.1087440,

p_mort_80_84, 0.1359920, 0.2039880,

p_mort_85_89, 0.2309360, 0.3464040,

p_mort_90_plus, 0.3642560, 0.5463840,

hr_mort_nyha1, 0.8000000, 1.2000000,

hr_mort_nyha2, 1.4400000, 2.1600000,

hr_mort_nyha34, 3.7600000, 5.6400000,

\#### Adverse events and SRT ####

p_ae_af, 0.0009360, 0.0014040,

p_srt_nyha34, 0.0269976, 0.0404964,

\#### Utilities ####

u_nyha1, 0.7280000, 1.0000000,

u_nyha2, 0.6800000, 1.0000000,

u_nyha34, 0.5600000, 0.8400000,

u_ae_grade34_af, 0.1520000, 0.2280000,

\#### Costs ####

c_aficamten_week, 165.1917404, 247.7876106,

c_metoprolol_week, 0.1867257, 0.2799410,

c_verapamil_week, 4.8318584, 7.2477876,

c_hcru_nyha1_annual, 1611.1489676, 2416.7241888,

c_hcru_nyha2_annual, 6397.6135693, 9596.4188791,

c_hcru_nyha34_annual, 15933.2802360, 23899.9203540,

c_srt_event, 793.0191740, 1189.5294985,

c_ae_af_event, 5444.5427729, 8166.8141593,

c_monitoring, 30.68,	46.02)

\## Run one-way sensitivity analysis

ndsa1 <- run_dsa(

res_mod2, dsa)

abc <- ndsa1$dsa

df2 <- subset(abc, .strategy_names == "TG")

df0 <- subset(abc, .strategy_names == "CG")

df20 <- data.frame(par=df2$.par_names,color=df2$.par_value, ICER=(df2$.cost-df0$.cost)/(df2$.effect-df0$.effect))

df20 <- df20 %>%

group_by(par) %>%

mutate( label = ifelse(color == min(color), "low", "high") )

df20 <- df20 %>%

mutate(ICER_adjusted = ICER - 29399.70)

df20 <- df20 %>%

mutate(color = ifelse(label == "high", "red", "blue"))

df_sorted20 <- df20 %>%

group_by(par) %>%

mutate(diff = abs(ICER - lag(ICER))) %>%

filter(!is.na(diff)) %>%

arrange(diff) %>%

ungroup()

\# Add the calculated difference to the original data frame and sort

df_sorted20 <- df20 %>%

inner_join(df_sorted20 %>% select(par, diff), by = "par") %>%

arrange(diff, par)

write.xlsx(df_sorted20, file="tornado_data_waning_after_week96_complete_loss_year3.xlsx")

\## Plot tornado diagram

source("plotting_functions.R")

options(scipen = 999)

tornado_plot <- plot_tornado(

df_sorted20 = df_sorted20,

minn = 15,

par = par,

ev = 22802.65,

Tornado_name_title = "One-way sensitivity analysis",

Tornado_name_x = "ICER ($/QALY)",

Tornado_size_y = 10,

Tornado_legend.position = c(0.85, 0.85),

x_min = 10000,

x_max = 60000,

x_by = 50000,

position_ev = 17000,

WTP = 29399.70,

color_low = "#4E79A7",

color_high = "#A23E48")

ggsave("tornado_plot_waning_after_week96_complete_loss_year3.JPG", plot = tornado_plot, units = "cm", width = 30, height = 18, dpi = 600)
